# Exploring and prioritizing content to include in a medication self-management toolkit for persons with spinal cord injury/dysfunction: A concept mapping approach

**DOI:** 10.1371/journal.pone.0310323

**Published:** 2024-10-31

**Authors:** Lauren Cadel, Rasha El-Kotob, Sander L. Hitzig, Lisa M. McCarthy, Shoshana Hahn-Goldberg, Tanya L. Packer, Chester H. Ho, Tejal Patel, Stephanie R. Cimino, Aisha K. Lofters, Sara J. T. Guilcher

**Affiliations:** 1 Leslie Dan Faculty of Pharmacy, University of Toronto, Toronto, ON, Canada; 2 Institute for Better Health, Trillium Health Partners, Mississauga, ON, Canada; 3 Temerty Faculty of Medicine, Rehabilitation Sciences Institute, University of Toronto, Toronto, ON, Canada; 4 Sunnybrook Research Institute, St. John’s Rehab Research Program, Sunnybrook Health Sciences Centre, Toronto, ON, Canada; 5 Temerty Faculty of Medicine, Department of Occupational Science and Occupational Therapy, University of Toronto, Toronto, ON, Canada; 6 Schlegel-University of Waterloo Research Institute of Aging, Waterloo, ON, Canada; 7 Department of Family and Community Medicine, University of Toronto, Toronto, ON, Canada; 8 Women’s College Research Institute, Toronto, ON, Canada; 9 OpenLab, University Health Network, Toronto, ON, Canada; 10 Schools of Occupational Therapy and Health Administration, Dalhousie University, Halifax, NS, Canada; 11 Department of Nursing, Umeå University, Umeå, Sweden; 12 Department of Clinical Neurosciences, Division of Physical Medicine & Rehabilitation, Foothills Medical Centre, Calgary, AB, Canada; 13 School of Pharmacy, University of Waterloo, Kitchener, ON, Canada; 14 Institute of Health Policy, Management and Evaluation, University of Toronto, Toronto, ON, Canada; University of Pretoria, SOUTH AFRICA

## Abstract

**Background:**

Adults with spinal cord injury/dysfunction (SCI/D) face challenges with medications they take to manage their secondary conditions (e.g., pain, urinary tract infections, autonomic dysreflexia). With many healthcare providers typically involved in care, there are additional challenges with care fragmentation and self-management. Prior research emphasized the desire for more support with medication self-management among this population.

**Objective:**

To explore what content should be included in a medication self-management resource (i.e., toolkit) for adults with SCI/D, as well as considerations for delivery from the perspectives of adults with SCI/D, caregivers, healthcare providers, and representatives from community organizations.

**Methods:**

A concept mapping study was conducted. Participants took part in one or more of three activities: brainstorming; sorting and rating; and mapping. Participants generated ideas about the content to include in a medication self-management toolkit. Participants sorted the statements into conceptual piles and assigned a name to each. All statements were rated on a five-point Likert-type scale on importance and realistic to include in the toolkit. Participants decided on the final cluster map, rearranged statements, and assigned a name to each cluster to create visual representations of the data.

**Results:**

Forty-four participants took part in this study. The final map contained eight clusters: 1) information-sharing and communication; 2) healthcare provider interactions and involvement; 3) peer and community connections; 4) supports and services for accessing prescription medications and medication information; 5) information on non-prescription medication and medication supplies; 6) safety and lifestyle considerations; 7) general medication information; and 8) practical information and strategies related to medication-taking. Safety and lifestyle considerations was rated as the most important and realistic to include in the toolkit.

**Conclusions:**

Given the limited tools to help adults with SCI/D with managing their medications, there is great potential to better support this population across all areas of medication self-management.

## Introduction

A spinal cord injury/dysfunction (SCI/D) occurs from damage to the spinal cord from either a traumatic spinal cord injury (TSCI; e.g., as a result of motor vehicle collision, fall) or non-traumatic spinal cord dysfunction (NTSCD; e.g., as a result of infection, spinal tumor). It is estimated that approximately 15.4 million people worldwide are living with SCI/D [1] and over 86,000 people in Canada are living with SCI/D [2]. In Canada, approximately 51% of individuals have a TSCI and 49% have a NTSCD. Among those with TSCI, the ratio of males to females is approximately three to one, while among those with NTSCD, the ratio of males to females is approximately three to two [2]. The average age at time of injury is higher among individuals with NTSCD (61 compared to 54 years old) [2].

Secondary conditions are common post-SCI/D due to deficits in motor, sensory, and autonomic functions [3]. Frequently experienced secondary conditions post-SCI/D include: pain, spasticity, neurogenic bladder and bowel, sexual dysfunction, cardiovascular complications, respiratory complications, pressure injuries, depression, and anxiety [4–8]. Secondary conditions can be acute or chronic and may also be episodic in nature. Secondary conditions may present individuals with SCI/D with numerous challenges such as functional impairments and interference with daily activities and thus, are often managed both pharmacologically and non-pharmacologically [9].

As a result of secondary conditions, medication use often increases substantially after experiencing SCI/D, with a prospective study by Jensen and colleagues reporting a three-time increase in medication use post-injury [10]. The use of multiple medications or polypharmacy (often defined as the use of five or more medications) is also common in this population [11–14]. While multiple medications may be appropriate due to the co-occurrence of secondary conditions, their use may also increase one’s risk for medication-related harm, including drug interactions and adverse drug events [11,14]. Given the diversity of secondary conditions, individuals with SCI/D often receive care from several different specialists; thus, increasing the risk of potential medication interactions and lack of a coordinated approach to medication management [15–17]. Among persons with SCI/D, a number of significant challenges managing multiple medications have been previously reported, which include but are not limited to: understanding and effectively integrating medications into daily routines, regulating emotions when dealing with complex medication regimens, managing medication side effects, and accessing and communicating with healthcare providers about their medications [18]. These challenges map onto several core aspects of medication self-management.

Medication self-management is the range of tasks, skills, and behaviours required to navigate the physical, social, and cognitive lifestyle factors, changes, and consequences inherent in taking, or choosing not to take, medications in everyday life (p.3) [19]. This includes the core tasks needed to have the knowledge, confidence, and skills to deal with medical, emotional, and role management, and solve problems, make decisions, seek supports, set and tailor goals, engage in activities, and participate in social interactions, as they relate to managing medications [19–22].

Previous qualitative research among persons with SCI/D highlighted the need for more support with medication management post-discharge from hospital or rehabilitation, specific to integrating medication-taking into ones’ daily routines, identifying individualized strategies for managing medications, and navigating a new identity that includes medications [18]. Further, a scoping review of medication self-management interventions for persons with TSCI by Cadel et al. identified limited tools and supports for this population [19]. Only three articles met the review’s inclusion criteria, and none comprehensively addressed medication self-management, as previously defined. Despite the limited studies, improvements in perceived knowledge and confidence and a reduction in pain medications were noted following the implementation of educational interventions. The authors of this scoping review highlighted the importance of future work to co-design and evaluate an intervention that addresses most, if not all, aspects of medication self-management.

Therefore, the objective of this study was to explore what content should be included in a medication self-management toolkit for adults with SCI/D, as well as considerations for the delivery of the toolkit content from the perspectives of adults with SCI/D, caregivers, healthcare providers, and representatives from community organizations.

## Methods

### Study design

This exploratory sequential mixed methods study used concept mapping, a collaborative approach that leverages participant-driven data collection and analysis [23]. Concept mapping consists of six key steps: preparation, brainstorming, sorting and rating, analysis, mapping and interpretation, and utilization. The qualitative portions of this mixed methods study were the brainstorming and sorting steps, and the quantitative portion was the rating step. Integration occurred during the analysis and mapping and interpretation steps.

### Step 1: Preparation

Preparation involved determining the focus through the development of the focal prompt (main question asked of participants), recruiting participants, and organizing logistics for the brainstorming sessions. Participants included adults with SCI/D, caregivers, healthcare providers, and representatives from community organizations. For inclusion, adults with SCI/D were required to have TSCI or NTSCD, be at least three months post-injury, and living in Canada (in the community or in an institution). Caregivers were also required to live in Canada and were currently providing, or have provided, support to an adult with SCI/D who was at least three months post-injury. Healthcare providers and representatives from community organizations were required to be currently providing, or have provided, health or social care services to adults with SCI/D and be employed in Canada. All participants were required to be at least 18 years of age and able to read and communicate in English. There were no specific exclusion criteria.

Participants were recruited between October 2022 and October 2023 using purposive and convenience sampling strategies [24]. Participants from previous projects who were interested in being involved in future research were contacted for participation. During our consent process, permission to contact participants for future research projects in which they qualify was sought. Therefore, we had a pool of potential participants that we could contact during recruitment. We also leveraged our research teams’ networks, partnerships with community organizations, and social media to support recruitment. Among all participants, we sought variation in age, sex, gender, and race; among adults with SCI/D, we sought variation in injury type; and among healthcare providers, we sought variation in profession. While we sought variation based on these characteristics, no eligible participants were excluded. The overall sample size (n = 44) was based on guidelines for concept mapping and aligned with previously conducted concept mapping studies [23,25].

### Step 2: Brainstorming–idea generation

In the brainstorming step, participants generated a list of statements in response to the following focal prompt: *if a resource (e*.*g*., *toolkit) was being developed to help persons with SCI/D manage their medications*, *what should it include*? A question guide was developed using concepts from the Behaviour Change Wheel [26]. Developed by Michie et al., the Behaviour Change Wheel aims to guide the development of behaviour change interventions. The interview guide was informed by the behaviour system hub of the framework, which includes capability, opportunity, and motivation for changing one’s behaviour. Brainstorming sessions were conducted synchronously using Zoom (version: 5.17.2) or telephone and asynchronously using the online concept mapping platform, groupwisdom™ (https://www.groupwisdom.tech/login) [27]. Synchronous sessions were conducted virtually as 60-minute, one-on-one interviews or focus groups. All sessions were led by the same facilitator (LC), audio-recorded, and the statements were recorded by a notetaker. The audio-recording was reviewed by the facilitator following each brainstorming session to ensure no statements were missed by the notetaker. Statements generated in both synchronous and asynchronous sessions were combined to create a master list of all statements.

The research team condensed the master list of statements by condensing duplicates and removing statements that did not answer the focal prompt. Through group discussion amongst the research team, the statements were reworded for clarity and consistency. They were then randomized, assigned a number from 1 to 79 and the final statement list was uploaded to groupwisdom™ for sorting and rating.

### Step 3: Sorting and rating

Participants then completed two independent tasks, sorting and rating. During the sorting task, participants sorted the final list of statements into conceptually similar piles and assigned a name to each pile. For the rating task, participants rated each statement on two five-point Likert-type scales, importance (i.e., not at all important to extremely important) and realistic (i.e., not at all realistic to extremely realistic). For importance, the rating question was how important, or not important, is it to have each statement in a toolkit? For realistic, the rating question was: how realistic, or not realistic, is it to integrate each statement into a toolkit? All participants completed both tasks on groupwisdom™, receiving help from the research team to navigate the software, as needed.

### Step 4: Concept mapping analysis

Using groupwisdom™, a series of analyses were conducted to create visual representations of the data. First, similarity matrices were created to show how many participants sorted each pair of statements together [28]. These matrices were then combined to create a total square similarity matrix, which was used as the input for the multidimensional scaling. A point map was created from the multidimensional scaling to display the relative distances between statements as an indicator of how frequently the statements were sorted together. A stress value was calculated to evaluate the goodness of fit between the similarity matrix data and the point map. Stress values between 0.205 and 0.365 are noted as adequate for concept mapping [28]. Hierarchical cluster analysis was then conducted to group statements together based on their relative distance on the point map, which creates potential cluster map solutions. Average bridging values (ranging from 0 to 1) were calculated for each cluster to identify if the statements fit with the other statements within the cluster or act as a “bridge” to link to other clusters on the map. To explain this further, statements with lower bridging values are more homogenous and representative of the meaning of the cluster in which they are contained. In comparison, statements with higher bridging values represent a more heterogenous cluster and may be connected in meaning to more than one cluster [29].

Research team members (LC, RE, SJTG) reviewed the cluster map solutions and chose two cluster maps, the 7-cluster and 8-cluster maps to present to participants in the mapping session. Following guidance from Kane and Trochim, these maps were selected by the research as the most appropriate representations of the data from their perspective, given the groupings of thematically similar statements.

### Step 5: Mapping and interpretation

The mapping session was a 90-minute, synchronous, group session conducted virtually on Zoom. All participants had taken part in brainstorming and/or sorting and rating. The session was facilitated by members of the research team (LC, RE, and SJTG). In the mapping session, participants were presented with the 7-cluster and 8-cluster map solutions, and the group discussed what map they wanted to represent the data. The participants were led through each cluster by reviewing all the statements and noting potential statements to be moved to a different cluster where they may fit better conceptually. After finalizing the statements in each cluster, participants assigned a label to each cluster to represent the statements contained within it.

Following mapping and interpretation, several additional maps and visual representations of the data were created, including: cluster rating maps, pattern matches, and go-zone plots.

Cluster rating maps display the cluster ratings as layers, with a greater number of layers representing a higher average rating. Pattern matches display average cluster ratings based on selected characteristics. Go-zone plots display the mean statement values on a bivariate XY graph, with the upper right quadrant of the graph containing the statements rated above average on both dimensions. These maps and visual representations were created following the mapping session to reflect changes made to the clusters by participants during the session. The purpose of these maps was to explore potential differences in importance and realistic ratings by participant demographics, which would help inform the development of specific content for different groups.

### Step 6: Utilization

The results from this concept mapping study will be used to inform the development and delivery of a medication self-management toolkit for adults with SCI/D. We will also share the findings at relevant conferences that includes researchers, individuals with lived experience, healthcare providers, and policymakers.

### Ethics

All participants provided written consent prior to taking part in this study. This study received ethics approval from the Research Ethics Boards of the University of Toronto (#42195) and the University of Alberta (#Pro00121103).

## Results

### Participant characteristics

Forty-four participants took part in this concept mapping study: 30 took part in brainstorming, 34 in sorting, 35 in rating, and 10 in mapping. See Table 1 for participant numbers and demographics across the concept mapping steps. Overall, we had representation from adults with SCI/D (n = 21), caregivers (n = 11), and healthcare providers (n = 12). Participants were from six provinces across Canada: Ontario (n = 26), Alberta (n = 7), British Columbia (n = 5), Manitoba (n = 2), Saskatchewan (n = 1), Nova Scotia (n = 1), and missing (n = 2). There were more participants with traumatic (n = 16) than non-traumatic injuries (n = 5). Also, more participants were of female (n = 27) than male sex (n = 17) and identified as cis-gendered women (n = 27) than cis-gendered men (n = 17). The majority of participants identified as White (n = 25), with some representation of individuals who identified as Black (n<5), East/ Southeast Asian (n<5), Indigenous (n<5), South Asian (n<5), or other/ missing (n<5).

**Table 1:** Participant numbers and demographics across the steps of concept mapping.

Demographics	Brainstorming (n = 30)*	Sorting (n = 34)	Rating (n = 35)	Mapping (n = 10)
***Participant Type***				
Adults with TSCI	12	15	16	6
Adults with NTSCD	5	<5	<5	<5
Caregivers	<5	7	7	0
Healthcare providers and organization representatives	9	9	9	<5
***Sex***				
Male	12	12	13	<5
Female	17	22	22	6
***Gender***				
Man	12	12	13	<5
Woman	17	22	22	6
***Age Group***				
18–29	<5	<5	<5	<5
30–39	<5	10	10	0
40–49	<5	5	5	<5
50–59	9	8	9	<5
60–69	8	7	7	<5
70–79	<5	<5	<5	0

Black	<5	<5	<5	0
East/ Southeast Asian	<5	<5	<5	0
Indigenous	<5	<5	<5	0
South Asian	<5	<5	<5	<5
White	17	20	21	8
Other	<5	<5	<5	<5

*missing participant demographics for one caregiver.

### Cluster map

A total of 684 statements were generated in the synchronous and asynchronous brainstorming sessions. These were condensed to a final list of 79 statements when duplicates and unrelated statements were removed by the research team. Using groupwisdom™, the statements were randomized and assigned a number from 1 to 79. This finalized statement list was sorted, rated, and mapped to create content maps. In the mapping session, participants selected the 8-cluster map as the final solution: (1) information-sharing and communication; (2) healthcare provider interactions and involvement; (3) peer and community connections; (4) supports and services for accessing prescription medications and medication information; (5) information on non-prescription medication and medication supplies; (6) safety and lifestyle considerations; (7) general medication information; and (8) practical information and strategies related to medication-taking.

### Cluster 1 –information-sharing and communication

Cluster 1 contained seven statements, with a bridging value of 0.23. Statements in this cluster related to the sharing of information about medications, including communicating with individuals who may be supporting medication management, strategies for self-advocating and empowering individuals to make decisions about their medications, strategies for getting medications from the pharmacy, and topics to discuss with the pharmacy team. The statement rated highest on importance and realistic was statement *(S)#25 –information on medications that is based on scientific evidence* (mean = 4.09, 3.83; respectively).

### Cluster 2 –healthcare provider interactions and involvement

Cluster 2 contained 12 statements, with a bridging value of 0.24. Statements related to strategies for communicating and building relationships with healthcare providers, information on the roles of different healthcare providers and how to contact them, information on healthcare settings with expertise in SCI/D, access to healthcare providers with expertise in SCI/D, and strategies for accessing new healthcare providers and navigating between different providers. The statement rated highest on importance was *S#77 –a list of specialized healthcare providers or care settings with expertise in medication management for SCI/D* (mean = 4.0). The statement rated highest on realistic was *S#11 –a list of common questions to ask healthcare providers about medications* (mean = 4.09).

### Cluster 3 –peer and community connections

Cluster 3 contained seven statements, with a bridging value of 0.47. These statements were related to connections and support of peers, including strategies for connecting with others, personal stories and profiles, and a forum for sharing information about medications. The statement rated highest on importance was *S#16 –strategies for connecting individuals with SCI/D to other people (e*.*g*., *peers and/or healthcare providers) who speak the same language for medication-related support* (mean = 3.94) and the statement rated highest on realistic was *S#38—an interactive online forum for sharing and receiving information about medications (e*.*g*., *information sessions*, *peer communication)* (mean = 3.40)

### Cluster 4—supports and services for accessing prescription medications and medication information

Cluster 4 contained eight statements, with a bridging value of 0.54. Statements in this cluster related to accessing prescription medications and medication-related information, such as financial considerations, supports when traveling, sources of medication information, prescribed narcotics, and a search function within the toolkit. The statement rated highest on importance within this cluster was *S#2—strategies for accessing affordable medications (e*.*g*., *generics*, *insurance plans)* (mean = 4.20). *S#43—a search function within the toolkit to look for specific information about medications* (mean = 3.94) was rated as most realistic.

### Cluster 5—information on non-prescription medication and medication supplies

Cluster 5 contained seven statements, with the highest bridging value of 0.59. The statements in this cluster were related to accessing non-prescription medications and supplies, including information on the role of non-prescription medications, strategies for accessing and using cannabis and other non-prescription medications, and information on where to purchase medication supplies. In terms of importance, statement *S#29—information on the role of non-prescription medications (e*.*g*., *natural health products*, *over-the-counter medications) when managing common secondary conditions* (mean = 3.71) was rated as most important. The statement rated highest on realistic was *S#53—visuals (e*.*g*., *pictures*, *videos*, *infographics) to present the information in the toolkit* (mean = 3.89).

### Cluster 6—safety and lifestyle considerations

Cluster 6 contained 16 statements, with the lowest bridging value of 0.12. The statements within this cluster were specific to safety and lifestyle considerations, such as: information on pain, side effects, medication interactions, considerations before starting a new medication, medication adjustments, medication dependency, strategies for coping with medication-taking, what do to in emergency situations, and weighing the pros and cons of taking medications. The statement rated highest on importance was *S#3—information on being aware of what medications are taken and what they are for* (mean = 4.46). The statement rated highest on realistic was *S#62—information on pain*, *including management of pain with medications* (mean = 4.14).

### Cluster 7—general medication information

Cluster 7 contained seven statements, with a bridging value of 0.21. Statements within this cluster were related to information on medications such as: standard versus off-label use, brand name versus generic medications, commonly used medications within the SCI/D population, and strategies for staying up-to-date on medications. The statement rated highest on importance was *S#12—information on how medications may affect people with SCI/D differently than the general population* (mean = 4.31) and *S#23—a fillable tracking sheet to complete information about medications (e*.*g*., *list of medications*, *reason*, *dosage*, *picture)* (mean = 3.89) was rated highest on realistic.

### Cluster 8—practical information and strategies related to medication-taking

Cluster 8 contained 15 statements, with a bridging value of 0.22. The statements in this cluster were related to strategies to facilitate medication-taking, including: organizing one’s home environment, assistive devices, tracking medications, reminders, adapting routines and daily activities, and increasing independence. The statement rated highest on importance and realistic was *S#8—information about medications that is easy to understand* (mean = 4.29, 3.94).

### Pattern matches

**Fig 1:**
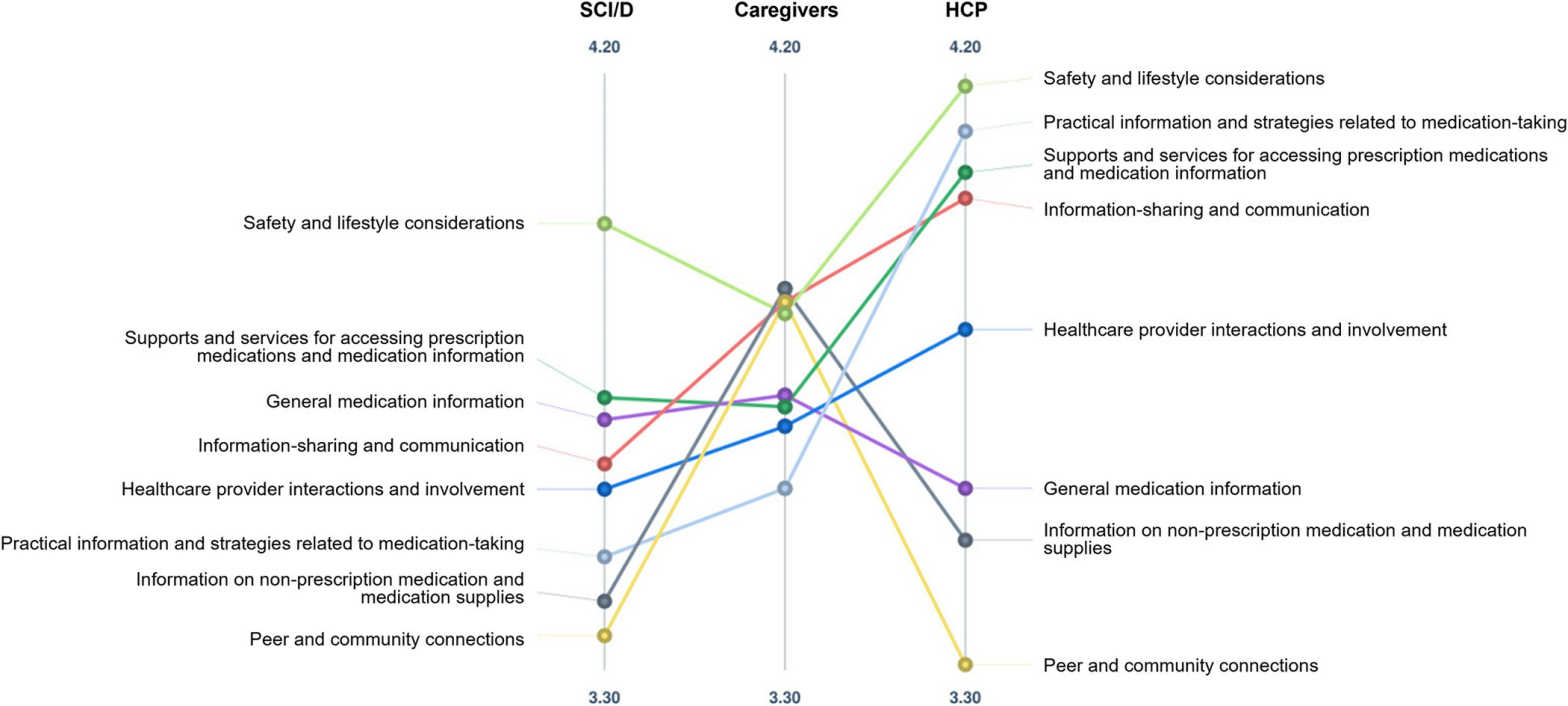
Pattern match diagram comparing importance ratings by participant type. Each point refers to a cluster, with the location on the vertical line representing the average cluster rating. Abbreviations: SCI/D = spinal cord injury/dysfunction; HCP = healthcare provider.

**Fig 2:**
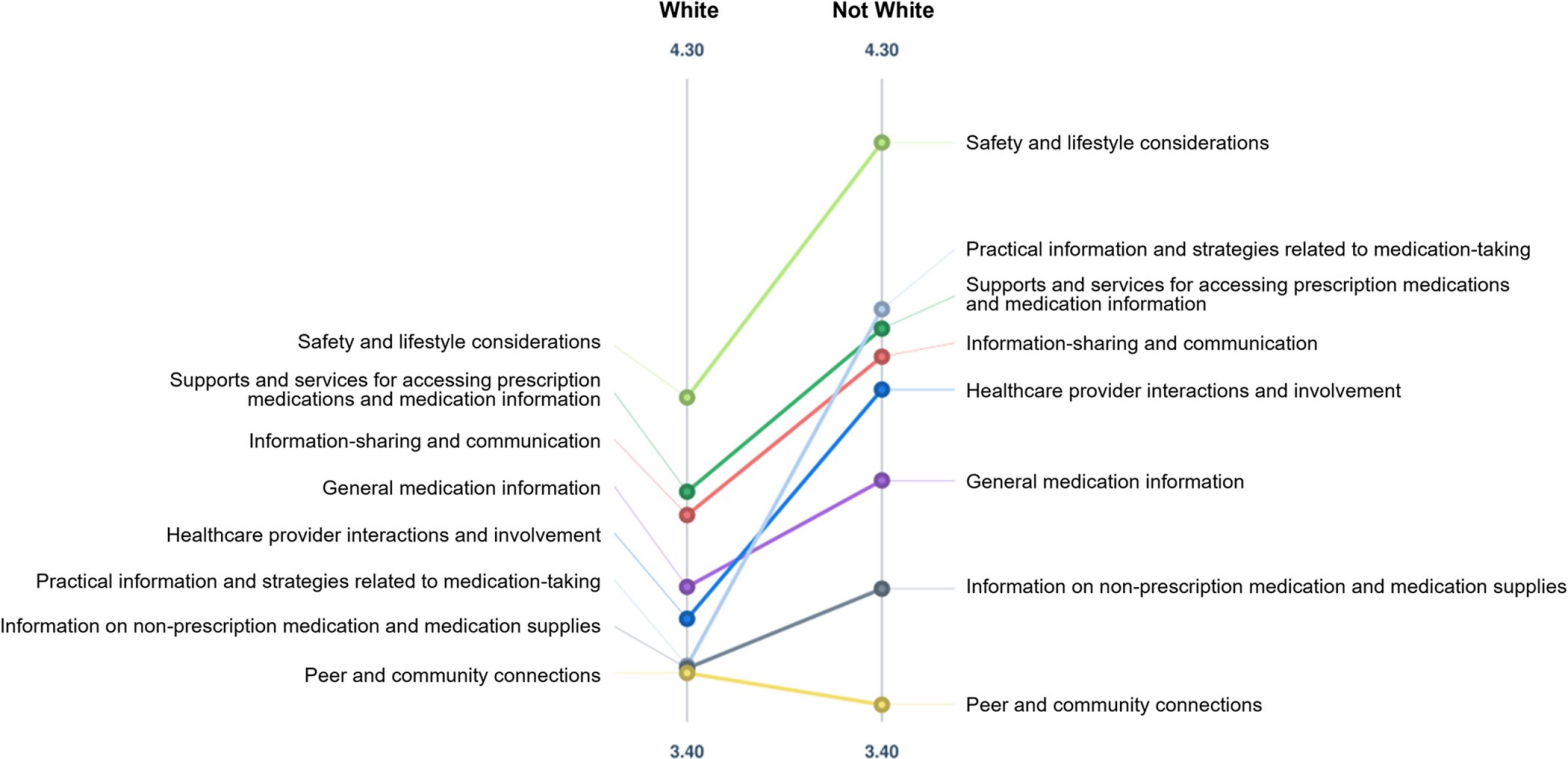
Pattern match diagram comparing importance ratings by race. Each point refers to a cluster, with the location on the vertical line representing the average cluster rating. Abbreviations: SCI/D = spinal cord injury/dysfunction; HCP = healthcare provider.

Pattern match diagrams represented average ratings for each cluster based on participants’ demographic characteristics. For participant type (Fig 1), visual differences in importance ratings were identified between adults with SCI/D, caregivers, and healthcare providers. Adults with SCI/D and healthcare providers rated *safety and lifestyle considerations* (mean = 3.98, 4.19; respectively) as the most important cluster, while caregivers rated *information on non-prescription medication and medication supplies* (mean = 3.88) as the most important. Caregivers rated *peer and community connections* (mean = 3.86) as the second most important cluster, but this was rated lowest on importance by both adults with SCI/D and healthcare providers (mean = 3.35, 3.30; respectively). Healthcare providers tended to rate all clusters higher on importance than adults with SCI/D. Participants who did not identify as White (identified as Black, East/ Southeast Asian, Indigenous, South Asian, or other) (Fig 2), rated all clusters except for *peer and community connections* higher on importance compared to participants who identified as White.

### Go-zone diagram

**Fig 3:**
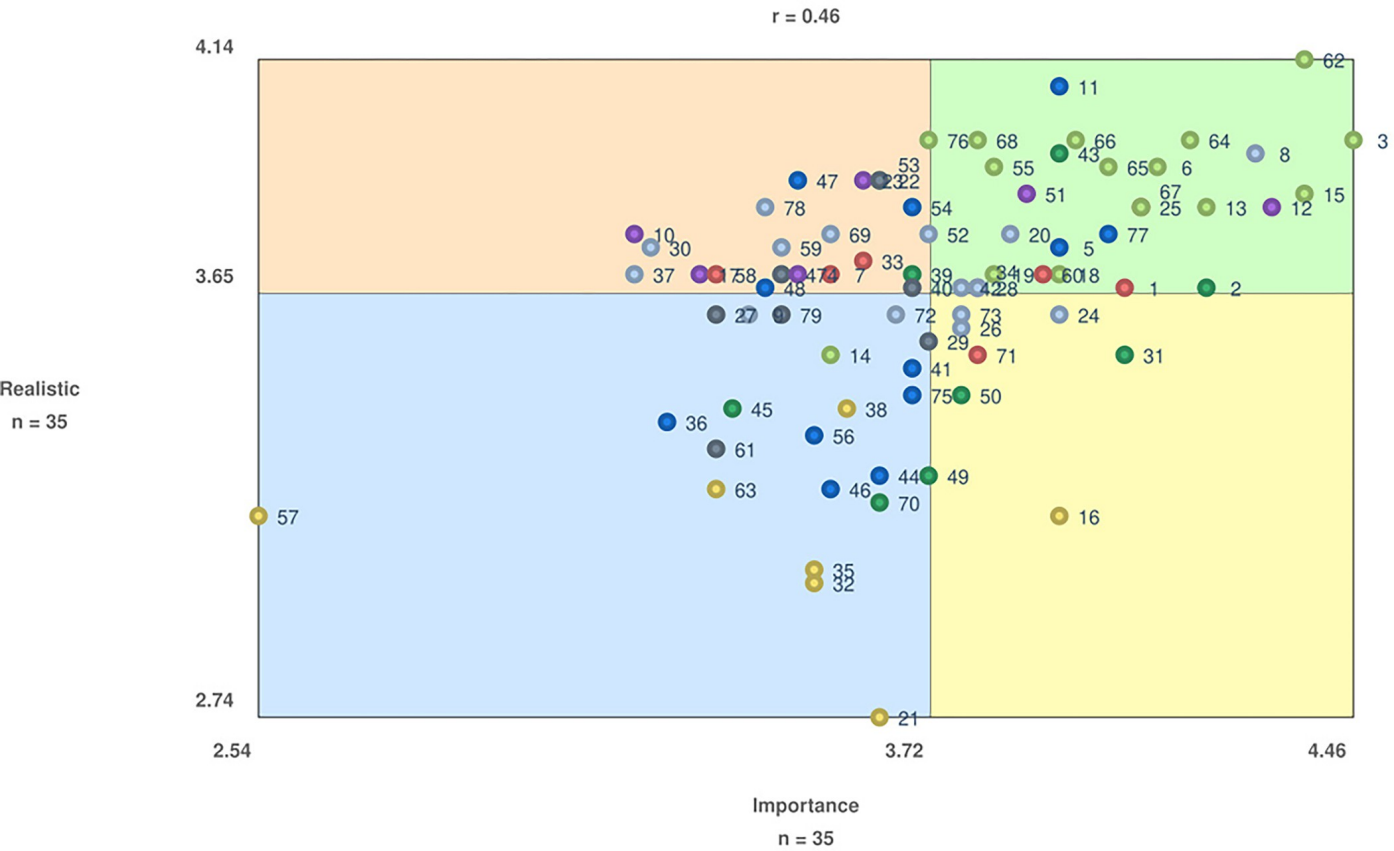
Go-zone diagram comparing importance and realistic ratings. Each numbered point refers to a statement (see Table 3 for the statement that corresponds to each numbered point). The ‘go-zone’ is the upper right quadrant (green), which contains the statements rated above average on both importance (mean = 3.72) and realistic (mean = 3.65) (see Table 2 for the statements contained in the go-zone).

A moderate correlation between importance and realistic ratings was identified (r = 0.46), as seen in Fig 3. Of the 79 total statements, 28 were included in the “go-zone”, meaning they were rated above average on both importance and realistic scales (Table 2). The two statements that rated highest on both dimensions were *62—information on pain*, *including management of pain with medications* (mean = 4.37, 4.14; respectively) and *3—information on being aware of what medications are taken and what they are for* (mean = 4.46, 3.97; respectively). The two statements rated lowest on both dimensions were *57—profiles of people with SCI/D who contributed to the toolkit* (mean = 2.54, 3.17; respectively) and *21—strategies for connecting individuals with SCI/D to other people (e*.*g*., *peers and/or healthcare providers) who are from a similar cultural background for medication-related support* (mean = 3.63, 2.74; respectively). Cluster 6, *safety and lifestyle considerations*, contained the most statements in the go-zone, with 13 of the 15 statements being rated above average on both importance and realistic.

**Table 2:** Go-zone statements, clusters, and average statement ratings.

Go Zone Statements	Cluster Number and Name	Mean Importance Rating (SD)	Mean Realistic Rating (SD)
1—Strategies for communicating with people who might help with medications (e.g., caregivers, personal support workers, coworkers)	Cluster 1—Information-sharing and communication	4.06 (1.00)	3.66 (1.14)
2—Strategies for accessing affordable medications (e.g., generics, insurance plans)	Cluster 4—Supports and services for accessing prescription medications and medication information	4.20 (0.72)	3.66 (1.08)
3—Information on being aware of what medications are taken and what they are for	Cluster 6—Safety and lifestyle considerations	4.46 (0.70)	3.97 (1.10)
5—Strategies for sharing medication-related information with healthcare providers	Cluster 2—Healthcare provider interactions and involvement	3.94 (0.91)	3.74 (1.01)
6—Information on things to consider when taking medications (e.g., with or without food)	Cluster 6—Safety and lifestyle considerations	4.11 (0.96)	3.91 (1.04)
8—Information about medications that is easy to understand	Cluster 8—Practical information and strategies related to medication-taking	4.29 (0.89)	3.94 (1.08)
11—A list of common questions to ask healthcare providers about medications	Cluster 2—Healthcare provider interactions and involvement	3.94 (0.94)	4.09 (0.85)
12—Information on how medications may affect people with SCI/D differently than the general population	Cluster 7—General medication information	4.31 (0.87)	3.83 (1.07)
13—Strategies for identifying, monitoring, and managing medication-related side effects	Cluster 6—Safety and lifestyle considerations	4.20 (0.83)	3.83 (0.92)
15—Information on steps to follow in case of accidental or emergency situations (e.g., missed dose, overdose, withdrawal)	Cluster 6—Safety and lifestyle considerations	4.37 (0.84)	3.86 (1.14)
18—Information on things to consider before starting a new medication	Cluster 6—Safety and lifestyle considerations	3.94 (0.87)	3.69 (1.02)
19—Strategies for weighing the pros and cons of taking a medication	Cluster 6—Safety and lifestyle considerations	3.83 (0.95)	3.69 (0.90)
20—Strategies for minimizing the impact of medication-taking on daily activities (e.g., timing of medications)	Cluster 8—Practical information and strategies related to medication-taking	3.86 (1.00)	3.77 (0.88)
25—Information on medications that is based on scientific evidence	Cluster 1—Information-sharing and communication	4.09 (0.82)	3.83 (1.01)
28—Strategies for staying updated about new medications for conditions related to SCI/D (e.g., pain, spasticity)	Cluster 7—General medication information	3.80 (0.99)	3.66 (0.91)
34—Strategies for tracking information about medications (e.g., medications, refills, side effects, symptoms, examples of tools)	Cluster 8—Practical information and strategies related to medication-taking	3.80 (0.99)	3.66 (1.00)
42—Strategies for helping with medication management when transitioning from hospital to home	Cluster 8—Practical information and strategies related to medication-taking	3.77 (1.14)	3.66 (1.00)
43—A search function within the toolkit to look for specific information about medications	Cluster 4—Supports and services for accessing prescription medications and medication information	3.94 (1.14)	3.94 (1.08)
51—A list of medications most commonly used by individuals with SCI/D	Cluster 7—General medication information	3.89 (0.96)	3.86 (1.03)
55—Information on antibiotic use and misuse (e.g., overuse, self-prescribing, prevention)	Cluster 6—Safety and lifestyle considerations	3.83 (0.95)	3.91 (0.89)
60—Strategies for self-advocating about medications across different care settings (e.g., inpatient acute hospital, inpatient rehabilitation, community)	Cluster 1—Information-sharing and communication	3.91 (1.01)	3.69 (0.96)
62—Information on pain, including management of pain with medications	Cluster 6—Safety and lifestyle considerations	4.37 (0.77)	4.14 (0.85)
64—Information on changing or stopping medications	Cluster 6—Safety and lifestyle considerations	4.17 (0.82)	3.97 (0.98)
65—Information on safety considerations of commonly used medications	Cluster 6—Safety and lifestyle considerations	4.03 (0.98)	3.91 (1.07)
66—Information about possibly becoming dependent on medications (e.g., laxatives)	Cluster 6—Safety and lifestyle considerations	3.97 (0.92)	3.97 (0.98)
67—Strategies for avoiding medication interactions (e.g., other medications, food, and beverages)	Cluster 6—Safety and lifestyle considerations	4.09 (0.92)	3.83 (0.89)
68—Information on different methods of taking medications (e.g., oral, injection, topical)	Cluster 6 –Safety and lifestyle considerations	3.80 (1.02)	3.97 (1.12)
77—A list of specialized healthcare providers or care settings with expertise in medication management for SCI/D	Cluster 2—Healthcare provider interactions and involvement	4.03 (0.92)	3.77 (1.00)

## Discussion

The purpose of this study was to explore what content to include in a toolkit to support medication self-management for adults with SCI/D, as well as to identify the most important and realistic content to include in the toolkit. In the brainstorming step, adults with SCI/D, caregivers, and healthcare providers generated nearly 700 ideas, which were synthesized into a final list of 79 statements. These statements were organized into eight clusters: (1) information-sharing and communication; (2) healthcare provider interactions and involvement; (3) peer and community connections; (4) supports and services for accessing prescription medications and medication information; (5) information on non-prescription medication and medication supplies; (6) safety and lifestyle considerations; (7) general medication information; and (8) practical information and strategies related to medication-taking. Key discussion points based on the findings of this study were the comprehensive nature of statements developed and the emphasis on safety and lifestyle considerations.

**Fig 4:**
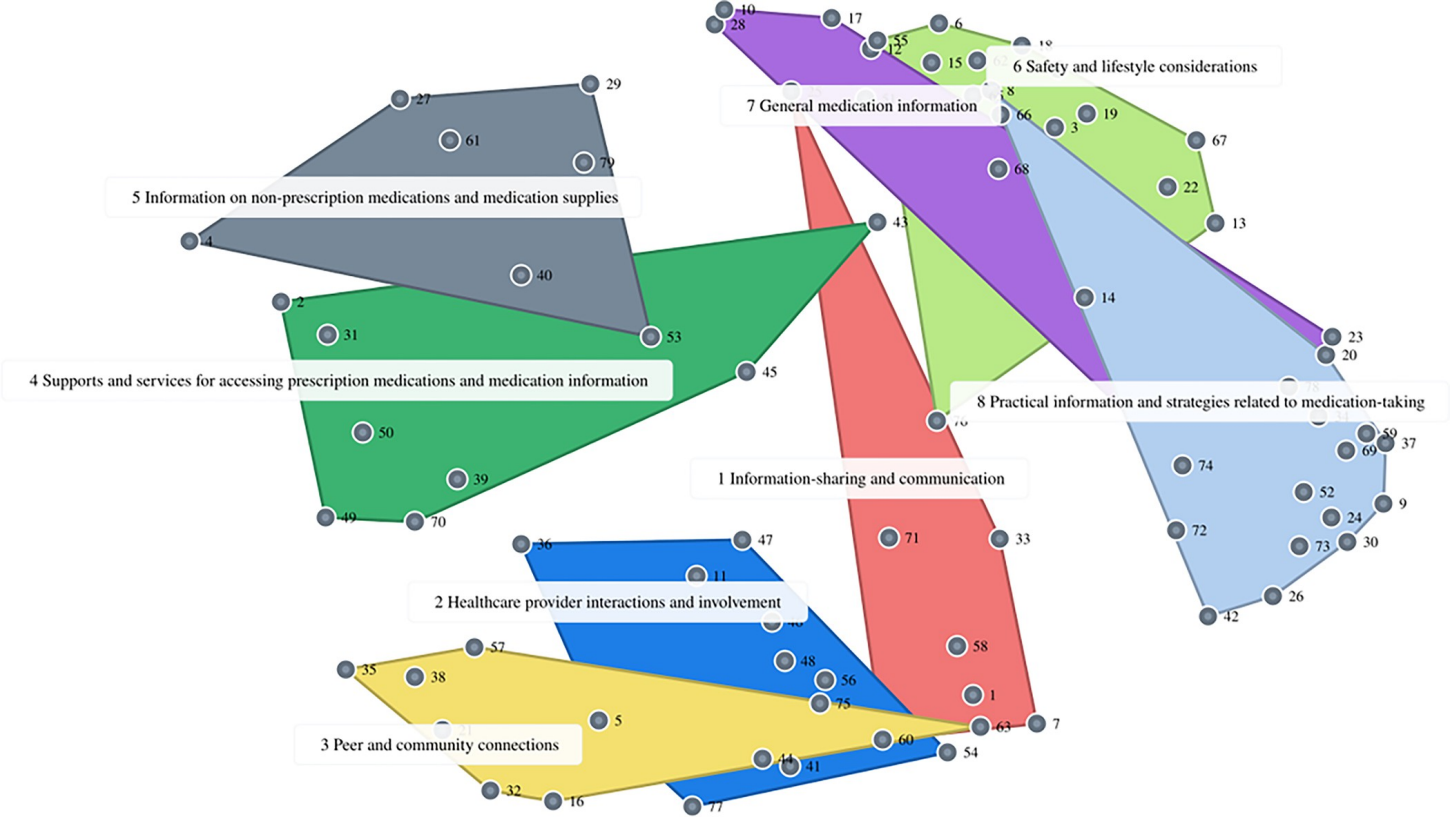
Concept map displaying 8-cluster map solution selected and adapted by participants (n = 10). Each numbered point refers to a statement (see Table 3 for numbered statements).

In this study, participants generated a wide range of ideas about the content to include in the toolkit reflecting the multi-dimensional aspects of medication-self management [18]. The final list of statements reflects the comprehensive and complex nature of medication self-management, by addressing a number of tasks, skills, and behaviours. We found that most statements were rated highly on both importance and realistic, with only two statements having a mean rating below neutral (moderately important or realistic). Given the comprehensiveness of the statements generated, along with the high ratings on both importance and realistic across all clusters, it is evident that participants valued aspects of medication self-management extending beyond medical management (e.g., taking medications). More specifically, statements touched on the three main tasks of self-management: medical, emotional, and role management, as well as the following skills: problem solving, decision making, support seeking, goal setting, engaging in activities, and participating in social situations [19–22]. The majority of clusters contained statements that aligned with multiple tasks and skills, which can be illustrated by the cluster map (Fig 4), with the overlay of multiple clusters.

**Table 3:** Cluster names, statements, and average statement ratings.

Cluster Number and Name	Statements	Mean Importance Rating^a^ (SD)	Mean Realistic Rating^b^ (SD)
Cluster 1—Information-sharing and communication Bridging value = 0.23	1—Strategies for communicating with people who might help with medications (e.g., caregivers, personal support workers, coworkers)	4.06 (1.00)	3.66 (1.14)
	7—Strategies for identifying people to help with managing medications (e.g., caregivers, personal support workers)	3.54 (0.98)	3.69 (1.23)
	25—Information on medications that is based on scientific evidence	4.09 (0.82)	3.83 (1.01)
	33—Information on medication-related topics to discuss with the pharmacy team (e.g., packaging, delivery, refills)	3.60 (0.98)	3.71 (1.23)
	58—Strategies for getting medications from the pharmacy (medication delivery, family/friends, accessible transit, arranging transportation)	3.34 (1.28)	3.69 (1.08)
	60—Strategies for self-advocating about medications across different care settings (e.g., inpatient acute hospital, inpatient rehabilitation, community)	3.91 (1.01)	3.69 (0.96)
	71—Strategies for empowering individuals to be actively involved in medication-related decisions (e.g., list of questions to ask self, do own research)	3.80 (0.99)	3.51 (1.12)
Cluster 2—Healthcare provider interactions and involvement Bridging value = 0.24	5—Strategies for sharing medication-related information with healthcare providers	3.94 (0.91)	3.74 (1.01)
	11—A list of common questions to ask healthcare providers about medications	3.94 (0.94)	4.09 (0.85)
	36—Strategies for communicating with healthcare providers about non-prescription medications (e.g., natural health products, over-the-counter medications)	3.26 (0.89)	3.37 (0.91)
	41—Strategies for fostering a good relationship with healthcare providers to support medication management	3.69 (0.93)	3.49 (0.98)
	44—Strategies for accessing a new healthcare provider for medication management (e.g., nurse practitioner, physician, pharmacist)	3.63 (0.94)	3.26 (1.01)
	46—Strategies for accessing public and private home care services to support medication-taking	3.54 (1.01)	3.23 (1.19)
	47—Information on medication-related topics to discuss with healthcare providers (e.g., physicians, nurses, pharmacists)	3.49 (0.95)	3.89 (1.02)
	48—Information on the roles and responsibilities of different healthcare providers (e.g., nurse practitioner, physician, pharmacist)	3.43 (1.07)	3.66 (1.14)
	54—A fillable sheet with healthcare providers’ contact information and when to contact them	3.69 (1.16)	3.83 (1.10)
	56—Information on the different ways to contact healthcare providers (e.g., email, in-person, virtual)	3.51 (0.89)	3.34 (1.06)
	75—Strategies for navigating multiple healthcare providers (e.g., nurse practitioner, physician, pharmacist), including how to switch between providers	3.69 (1.05)	3.43 (1.27)
	77—A list of specialized healthcare providers or care settings with expertise in medication management for SCI/D	4.03 (0.92)	3.77 (1.00)
Cluster 3—Peer and community connections Bridging value = 0.47	16—Strategies for connecting individuals with SCI/D to other people (e.g., peers and/or healthcare providers) who speak the same language for medication-related support	3.94 (0.76)	3.17 (1.20)
	21—Strategies for connecting individuals with SCI/D to other people (e.g., peers and/or healthcare providers) who are from a similar cultural background for medication-related support	3.63 (1.06)	2.74 (1.17)
	32—Strategies for connecting with peers about medications (e.g., how and where to access peer support, peer contact information)	3.51 (0.92)	3.03 (1.10)
	35—Personal stories from peers who can share their experiences with different medications (e.g., links to resources that have narratives)	3.51 (1.15)	3.06 (1.21)
	38—An interactive online forum for sharing and receiving information about medications (e.g., information sessions, peer communication)	3.57 (0.92)	3.40 (1.01)
	57—Profiles of people with SCI/D who contributed to the toolkit	2.54 (1.15)	3.17 (1.29)
	63—Strategies from peers for taking medications (e.g., reminder methods)	3.34 (1.19)	3.23 (1.14)
Cluster 4—Supports and services for accessing prescription medications and medication information Bridging value = 0.54	2—Strategies for accessing affordable medications (e.g., generics, insurance plans)	4.20 (0.72)	3.66 (1.08)
	31—Information on the financial costs of medications (e.g., dispensing fees, medication costs, packaging fees for blister packs)	4.06 (0.94)	3.51 (1.17)
	39—Strategies for accessing prescribed narcotics (e.g., opioids) from healthcare providers	3.69 (0.99)	3.69 (1.16)
	43—A search function within the toolkit to look for specific information about medications	3.94 (1.14)	3.94 (1.08)
	45—Strategies for accessing different sources of medication information (e.g., social media, internet, healthcare providers, peers)	3.37 (1.09)	3.40 (1.09)
	49—A list of pharmacies that are known for supporting individuals with SCI/D (based on positive feedback, medication delivery, accessibility)	3.71 (1.18)	3.26 (1.31)
	50—Voice activation technology so individuals can listen to the information in the toolkit	3.77 (1.21)	3.43 (1.07)
	70—Strategies for accessing medication supports and services if traveling to other regions	3.63 (1.14)	3.20 (1.28)
Cluster 5—Information on non-prescription medication and medication supplies Bridging value = 0.59	4—Strategies for accessing cannabis and cannabis-related products	3.46 (1.15)	3.69 (0.99)
	27—Strategies for using/taking cannabis and cannabis-related products	3.34 (1.00)	3.60 (0.95)
	29—Information on the role of non-prescription medications (e.g., natural health products, over-the-counter medications) when managing common secondary conditions	3.71 (0.99)	3.54 (0.92)
	40—Information on where to buy common medication-related supplies (e.g., dosettes, syringes)	3.69 (1.05)	3.66 (1.16)
	53—Visuals (e.g., pictures, videos, infographics) to present the information in the toolkit	3.63 (1.19)	3.89 (1.11)
	61—Strategies for accessing non-prescription medications (e.g., natural health products, over-the-counter medications)	3.34 (0.91)	3.31 (1.05)
	79—Strategies for taking non-prescription medications (e.g., natural health products, over-the-counter medications)	3.46 (1.17)	3.60 (1.01)
Cluster 6—Safety and lifestyle considerations Bridging value = 0.12	3—Information on being aware of what medications are taken and what they are for	4.46 (0.70)	3.97 (1.10)
	6—Information on things to consider when taking medications (e.g., with or without food)	4.11 (0.96)	3.91 (1.04)
	13—Strategies for identifying, monitoring, and managing medication-related side effects	4.20 (0.83)	3.83 (0.92)
	14—Information about how assistance with medication management may change over time post-injury (e.g., changes in dexterity, caregiving support)	3.54 (1.04)	3.51 (1.04)
	15—Information on steps to follow in case of accidental or emergency situations (e.g., missed dose, overdose, withdrawal)	4.37 (0.84)	3.86 (1.14)
	18—Information on things to consider before starting a new medication	3.94 (0.87)	3.69 (1.02)
	19—Strategies for weighing the pros and cons of taking a medication	3.83 (0.95)	3.69 (0.90)
	22—Information on lifestyle considerations when taking medications (e.g., alcohol use, social activities)	3.63 (1.00)	3.89 (0.99)
	55—Information on antibiotic use and misuse (e.g., overuse, self-prescribing, prevention)	3.83 (0.95)	3.91 (0.89)
	62—Information on pain, including management of pain with medications	4.37 (0.77)	4.14 (0.85)
	64—Information on changing or stopping medications	4.17 (0.82)	3.97 (0.98)
	65—Information on safety considerations of commonly used medications	4.03 (0.98)	3.91 (1.07)
	66—Information about possibly becoming dependent on medications (e.g., laxatives)	3.97 (0.92)	3.97 (0.98)
	67—Strategies for avoiding medication interactions (e.g., other medications, food, and beverages)	4.09 (0.92)	3.83 (0.89)
	68—Information on different methods of taking medications (e.g., oral, injection, topical)	3.80 (1.02)	3.97 (1.12)
	76—Strategies for how to cope with the mental/emotional aspects of taking medications post-injury (e.g., feeling overwhelmed, accepting medications)	3.71 (1.10)	3.97 (0.95)
Cluster 7—General medication information Bridging value = 0.21	10—Information on the difference between brand name and generic medications	3.20 (0.96)	3.77 (0.97)
	12—Information on how medications may affect people with SCI/D differently than the general population	4.31 (0.87)	3.83 (1.07)
	17—Information on the difference between standard use and off-label use of medication	3.31 (0.87)	3.69 (0.93)
	23—A fillable tracking sheet to complete information about medications (e.g., list of medications, reason, dosage, picture)	3.60 (1.14)	3.89 (1.13)
	28—Strategies for staying updated about new medications for conditions related to SCI/D (e.g., pain, spasticity)	3.80 (0.99)	3.66 (0.91)
	51—A list of medications most commonly used by individuals with SCI/D	3.89 (0.96)	3.86 (1.03)
	74—Information on apps that are available to support medication management	3.49 (1.15)	3.69 (1.05)
Cluster 8—Practical information and strategies related to medication-taking Bridging value = 0.22	8—Information about medications that is easy to understand	4.29 (0.89)	3.94 (1.08)
	9—Strategies for organizing the home to help with taking medications (e.g., table over bed, medication organizers in different rooms)	3.40 (0.98)	3.60 (1.19)
	20—Strategies for minimizing the impact of medication-taking on daily activities (e.g., timing of medications)	3.86 (1.00)	3.77 (0.88)
	24—Information on different tools (e.g., assistive devices) that enable people with SCI/D to take their medications	3.94 (1.00)	3.60 (1.17)
	26—Strategies for assisting with dexterity challenges when taking medication	3.77 (0.94)	3.57 (1.17)
	30—Strategies for taking medications when out of the house (e.g., small pill container, taking food/water)	3.23 (1.09)	3.74 (1.12)
	34—Strategies for tracking information about medications (e.g., medications, refills, side effects, symptoms, examples of tools)	3.80 (0.99)	3.66 (1.00)
	37—A checklist summarizing tasks to support taking medications	3.20 (1.13)	3.69 (1.30)
	42—Strategies for helping with medication management when transitioning from hospital to home	3.77 (1.14)	3.66 (1.00)
	52—Strategies for remembering to take medications (e.g., alarms, home care, blister packs)	3.71 (1.20)	3.77 (1.09)
	59—Strategies for integrating medication-taking as part of routine (e.g., taking with meals, before bed)	3.46 (1.17)	3.74 (0.95)
	69—Strategies for taking multiple medications throughout the day (e.g., creating a schedule, using an alarm or app)	3.54 (1.04)	3.77 (1.19)
	72—Strategies for adapting daily routines if usual support for medication-taking is not available (e.g., support persons, tools)	3.66 (1.24)	3.60 (1.06)
	73—Strategies for increasing independence in taking medications based on abilities (e.g., level and completeness of injury)	3.77 (1.11)	3.60 (1.01)
	78—Information on different ways to organize medications (e.g., blister packs, dosettes)	3.43 (1.04)	3.83 (1.18)

^a^Importance rating scale: 1 = not at all important; 2 = slightly important; 3 = moderately important; 4 = very important; 5 = extremely important.

^b^Realistic rating scale: 1 = not at all realistic; 2 = slightly realistic; 3 = moderately realistic; 4 = very realistic; 5 = extremely realistic.

Statements highlighted in blue were the highest rated statements in the cluster and statements highlighted in yellow were the lowest rated statements in the cluster.

With limited tools to help adults with SCI/D with managing their medications, there is great potential to better support this population across all areas of medication self-management [19]. For example, a recent scoping review identified three medication self-management interventions for adults with TSCI–a mobile app for general self-management post-SCI, an educational intervention to improve knowledge and confidence pertaining to medication management, and an educational intervention designed to improve knowledge and reduce pain medication use [19]. Two of these tools included medication self-management as a component of a larger intervention or educational program, rather than the primary focus. Despite this, some improvements were identified with improved knowledge and confidence related to bowel self-management and medication management from baseline to completion. Additionally, a reduction in pain medication use was also noted. However, the improvements in medication management knowledge and confidence were not maintained at the 60-day follow-up. Medications have been described to have a major impact on individuals with SCI/D; as such, a resource that specifically targets medication self-management and maintains improved outcomes over time should exist to provide adequate support for this population.

To support the sustainability of outcomes, focusing on domains identified by participants is key. *Safety and lifestyle considerations* was rated by participants as the most important and most realistic cluster to include in the toolkit. Prior research has highlighted both lifestyle and safety considerations related to medications in this population [14,18,30–33]. Cadel and colleagues’ qualitative study described the disruptive nature of medications and the interference of both complex medication regimens and medication side effects among community-dwelling adults with SCI/D engagement in meaningful daily activities [18]. Everall and colleagues compared perspectives of adults with SCI/D and healthcare providers on medications, noting that participants described the benefit of medications in managing secondary conditions, which facilitated involvement in daily activities [30]. Both Cadel and Everall also described safety concerns discussed by participants, especially around the long-term use of medications [18,30]. Several additional studies have also explored issues of medication safety by examining polypharmacy, adverse drug events, and medication-related problems in this population [11,14,31–33].

Examining medication-related problems among SCI/D, Patel and colleagues identified 34 events in a cohort of 19 individuals over an eight-month period [32]. Specific to oxycodone use, Barrera-Chacon et al. conducted a three-month prospective observational study and found that 54% of participants with TSCI experienced at least one medication-related adverse event [33]. There are high rates of polypharmacy among adults with SCI/D [11–14,32], and with this, there are associated safety concerns given the increased risk of adverse drug events. For example, individuals with SCI/D with polypharmacy are significantly more likely to experience a medication-related problem or adverse drug event than those without SCI/D [14,31]. Medication safety and long-term use of medications among adults with SCI/D is not a new topic, and has been previously discussed by both Høgholen et al. [34] and Cadel et al., [18] as they concluded that there is a need for more education to ensure persons with SCI/D feel safe with their regimen. As such, a toolkit designed for medication self-management has the potential to fill a critical gap by addressing lifestyle and safety considerations related to medications.

Toolkits are commonly used within healthcare for education and the promotion of skills and behaviour change [35–38]. Concept mapping has been used previously as a method for developing toolkits [39–42]. For example, Abelson and colleagues used a modified concept mapping approach to inform the development of a toolkit to support the evaluation of patient, caregiver, and family engagement in health system planning, design, and decision making [40]. Similarly, Goff et al. conducted a concept mapping study to co-design a web-based self-management toolkit for adults with knee osteoarthritis [42]. This study identified the educational priorities of people with knee osteoarthritis, as well as their perceived importance and confidence of the priorities. Concept mapping is beneficial for intervention and toolkit development because of the involvement of end-users, and it allows researchers to understand and prioritize the information received.

In our study, participants rated all statements relatively highly, with only two statements having ratings below moderate importance and realistic. In comparing ratings by demographic characteristics (e.g., age, sex, gender, participant type, race), we found that statements were rated highly regardless of participant characteristics. We noticed some visual differences in importance ratings based on participant type (adults with SCI/D, caregivers, healthcare providers, with adults and healthcare providers rating clusters in a similar order compared to caregivers) and race (White participants tended to rate clusters lower on importance compared to non-White participants). This presents an area to further explore in future research, by examining perspectives on content and usability, to ensure the toolkit is applicable to a diverse SCI/D population.

### Limitations and strengths

Study limitations included fewer caregiver participants, and those with non-traumatic injuries, and a sample that was not as racially diverse as anticipated. We did not have any caregiver participants in the mapping session, so the co-creation of the maps lacked this perspective. Racial diversity was also limited, despite recruitment efforts that strived to achieve this, as there was a higher percentage of White participants across all steps of concept mapping. However, this was especially relevant in the mapping session with only two participants who did not identify as White. Despite this lack of racial diversity and greater proportion of White individuals, it does align with other North American studies on racial disparities among adults with SCI/D, which also reported a greater proportion of White participants [43–45]. Across all stages, we had fewer participants with non-traumatic injuries than traumatic injuries. All data should be interpreted with these considerations in mind. It is important for future research to seek more participant, as targeted resources may need to be developed based on race and injury type. Despite these limitations, we included adults with SCI/D, caregivers, and healthcare providers to gather a wide range of perspectives on content to include in the toolkit. A key strength of this research was the involvement of persons with lived experience of a SCI/D in the co-development of content. Additionally, concept mapping uses a highly participatory approach to data collection and analysis. As such, future programs and interventions can focus on the most important areas of medication self-management identified by the target population.

### Areas for future research

These results from this concept mapping study will be used to inform the development of a medication self-management toolkit for adults with SCI/D across Canada. Content will be developed and revised. In subsequent phases of this research, the co-developed toolkit will undergo revisions through cognitive interviews. A pilot evaluation will then be conducted to assess the feasibility, acceptability, and appropriateness of the toolkit, as well as its preliminary effectiveness in improving aspects of medication self-management (e.g., knowledge, self-efficacy). While this research and planned next steps focus on adults with SCI/D, there is an opportunity to explore supporting roles for medication self-management. For example, identifying how healthcare providers could support education around medication self-management while individuals are in-hospital or rehabilitation to facilitate the transition to community. Additionally, understanding how caregivers can learn about the values and perspectives of adults with SCI/D to support the variety of tasks, skills, and behaviours associated with medication self-management.

## Conclusions

With the involvement of adults with SCI/D, caregivers, and healthcare providers, this study explored content to include in a medication self-management toolkit for adults with SCI/D. Seventy-nine statements were contained within eight clusters. The safety and lifestyle considerations cluster was perceived by participants as the most important and realistic to include in the toolkit. This study highlights areas of future research, including future steps in the co-development of this medication self-management toolkit for adults with SCI/D.

## References

[1] World Health Organization. Spinal Cord Injury Geneva, Switzerland: World Health Organization; 2024 [August 2024]. Available from: https://www.who.int/news-room/fact-sheets/detail/spinal-cord-injury#:~:text=Global%20estimates%20suggest%20that%20in,YLDs%20attributed%20to%20this%20demographic.

[2] Praxis Spinal Cord Institute. Rick Hansen Spinal Cord Injury Registry: A look at spinal cord injury in Canada in 2020. Vancouver, British Columbia, Canada: Praxis Spinal Cord Institute, 2022.

[3] Hou S, Rabchevsky AG. Autonomic consequences of spinal cord injury. Compr Physiol. 2014;4(4):1419–53. Epub 2014/11/28. doi: 10.1002/cphy.c130045 . 25428850

[4] Hagen EM. Acute complications of spinal cord injuries. World J Orthop. 2015;6(1):17–23. doi: 10.5312/wjo.v6.i1.17 PMC4303786. 25621207 PMC4303786

[5] Sezer N, Akkuş S, Uğurlu FG. Chronic complications of spinal cord injury. World J Orthop. 2015;6(1):24–33. doi: 10.5312/wjo.v6.i1.24 . 25621208 PMC4303787

[6] New PW. Secondary conditions in a community sample of people with spinal cord damage. J Spinal Cord Med. 2016;39(6):665–70. Epub 2016/02/24. doi: 10.1080/10790268.2016.1138600 ; PubMed Central PMCID: PMC5137565. 26899984 PMC5137565

[7] Strøm V, Månum G, Arora M, Joseph C, Kyriakides A, Le Fort M, et al. Physical Health Conditions in Persons with Spinal Cord Injury Across 21 Countries Worldwide. Journal of rehabilitation medicine. 2022;54:jrm00302. Epub 2022/06/10. doi: 10.2340/jrm.v54.2040 ; PubMed Central PMCID: PMC9272839. 35678293 PMC9272839

[8] Richardson A, Samaranayaka A, Sullivan M, Derrett S. Secondary health conditions and disability among people with spinal cord injury: A prospective cohort study. J Spinal Cord Med. 2021;44(1):19–28. Epub 2019/03/19. doi: 10.1080/10790268.2019.1581392 ; PubMed Central PMCID: PMC7919890. 30882288 PMC7919890

[9] Cadel L, DeLuca C, Hitzig SL, Packer TL, Lofters AK, Patel T, et al. Self-management of pain and depression in adults with spinal cord injury: A scoping review. The journal of spinal cord medicine. 2020;43(3):280–97. doi: 10.1080/10790268.2018.1523776 30335601 PMC7241513

[10] Jensen EK, Biering-Sørensen F. Medication before and after a spinal cord lesion. Spinal Cord. 2014;52(5):358–63. doi: 10.1038/sc.2014.20 24614857

[11] Cadel L, Everall AC, Hitzig SL, Packer TL, Patel T, Lofters A, et al. Spinal cord injury and polypharmacy: a scoping review. Disability and rehabilitation. 2020;42(26):3858–70. doi: 10.1080/09638288.2019.1610085 31068029

[12] Guilcher SJT, Hogan M-E, Calzavara A, Hitzig SL, Patel T, Packer T, et al. Prescription drug claims following a traumatic spinal cord injury for older adults: a retrospective population-based study in Ontario, Canada. Spinal Cord. 2018;56(11):1059–68. doi: 10.1038/s41393-018-0174-z 30065350 PMC6218396

[13] Guilcher SJT, Hogan M-E, McCormack D, Calzavara AJ, Hitzig SL, Patel T, et al. Prescription medications dispensed following a nontraumatic spinal cord dysfunction: a retrospective population-based study in Ontario, Canada. Spinal Cord. 2021;59(2):132–40. doi: 10.1038/s41393-020-0511-x 32665709

[14] Kitzman P, Cecil D, Kolpek JH. The risks of polypharmacy following spinal cord injury. The Journal of Spinal Cord Medicine. 2017;40(2):147–53. doi: 10.1179/2045772314Y.0000000235 24970339 PMC5430470

[15] McMillan C, Milligan J, Hillier LM, Bauman C, Donaldson L, Lee J. Why do challenges still exist in primary care for patients with spinal cord injury? Exploring the medical model through a social disability lens. Canadian Journal of Disability Studies. 2020;9(4):111–37. doi: 10.15353/cjds.v9i4.671

[16] Ho CH. Primary care for persons with spinal cord injury—not a novel idea but still under-developed. J Spinal Cord Med. 2016;39(5):500–3. Epub 2016/07/28. doi: 10.1080/10790268.2016.1182696 ; PubMed Central PMCID: PMC5020592. 27463240 PMC5020592

[17] Green JL, Hawley JN, Rask KJ. Is the number of prescribing physicians an independent risk factor for adverse drug events in an elderly outpatient population? Am J Geriatr Pharmacother. 2007;5(1):31–9. Epub 2007/07/05. doi: 10.1016/j.amjopharm.2007.03.004 . 17608245

[18] Cadel L, Hitzig SL, Packer TL, Patel T, Lofters AK, Thompson A, et al. Spinal cord injury/dysfunction and medication management: a qualitative study exploring the experiences of community-dwelling adults in Ontario, Canada. Disability and Rehabilitation. 2022;44(1):24–33. doi: 10.1080/09638288.2020.1756000 32362182

[19] Cadel L, Cimino SR, Bradley-Ridout G, Hitzig SL, Packer TL, McCarthy LM, et al. A scoping review of medication self-management intervention tools to support persons with traumatic spinal cord injury. PLOS ONE. 2023;18(4):e0284199. doi: 10.1371/journal.pone.0284199 37079514 PMC10118177

[20] Audulv Å, Ghahari S, Kephart G, Warner G, Packer TL. The Taxonomy of Everyday Self-management Strategies (TEDSS): A framework derived from the literature and refined using empirical data. Patient Education and Counseling. 2019;102(2):367–75. doi: 10.1016/j.pec.2018.08.034 30197252

[21] Lorig KR, Holman HR. Self-management education: History, definition, outcomes, and mechanisms. Annals of Behavioral Medicine. 2003;26(1):1–7. doi: 10.1207/S15324796ABM2601_01 12867348

[22] Cadel L, Cimino SR, Bradley-Ridout G, Hitzig S, Packer T, McCarthy L, et al. A scoping review of medication self-management interventions for persons with traumatic spinal cord injury. PLOS One [under review]. 2023. 10.1371/journal.pone.0284199 PMC10118177 37079514

[23] Kane M, Trochim WMK. Concept mapping for planning and evaluation. Thousand Oaks, California: Sage Publications; 2007.

[24] Collins KMT, Onwuegbuzie AJ, Jiao QG. A Mixed Methods Investigation of Mixed Methods Sampling Designs in Social and Health Science Research. Journal of Mixed Methods Research. 2007;1(3):267–94. doi: 10.1177/1558689807299526

[25] Donnelly JP. A systematic review of concept mapping dissertations. Eval Program Plann. 2017;60:186–93. Epub 2016/10/04. doi: 10.1016/j.evalprogplan.2016.08.010 . 27693034

[26] Michie S, van Stralen MM, West R. The behaviour change wheel: A new method for characterising and designing behaviour change interventions. Implementation Science. 2011;6(1):42. doi: 10.1186/1748-5908-6-42 21513547 PMC3096582

[27] groupwisdom™ ©2023 Concept Systems Inc. Ithaca, NY: Concept Systems Incorporated; 2023. Available from: https://groupwisdom.com/.

[28] Kane M, Trochim WMK. Concept mapping for planning and evaluation. Thousand Oaks, CA, US: Sage Publications, Inc; 2007.

[29] Laustsen CE, Westergren A, Petersson P, Haak M. Conceptualizing researchers’ perspectives on involving professionals in research: a group concept mapping study. Health Res Policy Syst. 2021;19(1):39. Epub 2021/03/20. doi: 10.1186/s12961-021-00685-2 ; PubMed Central PMCID: PMC7977251. 33736671 PMC7977251

[30] Everall AC, Cadel L, Lofters AK, Packer TL, Hitzig SL, Patel T, et al. An exploration of attitudes and preferences towards medications among healthcare providers and persons with spinal cord injury/dysfunction: a qualitative comparison. Disability and rehabilitation. 2022;44(8):1252–9. doi: 10.1080/09638288.2020.1799249 32755402

[31] Hand BN, Krause JS, Simpson KN. Polypharmacy and adverse drug events among propensity score matched privately insured persons with and without spinal cord injury. Spinal Cord. 2018;56(6):591–7. doi: 10.1038/s41393-017-0050-2 29362505 PMC8033691

[32] Patel T, Milligan J, Lee J. Medication-related problems in individuals with spinal cord injury in a primary care-based clinic. J Spinal Cord Med. 2017;40(1):54–61. Epub 2015/10/09. doi: 10.1179/2045772315Y.0000000055 ; PubMed Central PMCID: PMC5376141. 26446538 PMC5376141

[33] Barrera-Chacon JM, Mendez-Suarez JL, Jáuregui-Abrisqueta ML, Palazon R, Barbara-Bataller E, García-Obrero I. Oxycodone improves pain control and quality of life in anticonvulsant-pretreated spinal cord-injured patients with neuropathic pain. Spinal Cord. 2011;49(1):36–42. doi: 10.1038/sc.2010.101 20820176

[34] Høgholen H, Storhaug A, Kvernrød K, Kostovski E, Viktil KK, Mathiesen L. Use of medicines, adherence and attitudes to medicines among persons with chronic spinal cord injury. Spinal Cord. 2018;56(1):35–40. doi: 10.1038/sc.2017.95 28853448

[35] Barac R, Stein S, Bruce B, Barwick M. Scoping review of toolkits as a knowledge translation strategy in health. BMC Medical Informatics and Decision Making. 2014;14(1):121. doi: 10.1186/s12911-014-0121-7 25539950 PMC4308831

[36] Bennett MI, Mulvey MR, Campling N, Latter S, Richardson A, Bekker H, et al. Self-management toolkit and delivery strategy for end-of-life pain: the mixed-methods feasibility study. Health Technol Assess. 2017;21(76):1–292. doi: 10.3310/hta21760 . 29265004 PMC5757185

[37] Prochnow JA, Meiers SJ, Scheckel MM. Improving Patient and Caregiver New Medication Education Using an Innovative Teach-back Toolkit. Journal of Nursing Care Quality. 2019;34(2). doi: 10.1097/NCQ.0000000000000342 30198943

[38] van de Hei SJ, Dierick BJH, Aarts JEP, Kocks JWH, van Boven JFM. Personalized Medication Adherence Management in Asthma and Chronic Obstructive Pulmonary Disease: A Review of Effective Interventions and Development of a Practical Adherence Toolkit. The Journal of Allergy and Clinical Immunology: In Practice. doi: 10.1016/j.jaip.2021.05.025 34111571

[39] Blake H, Greaves S, Abbott-Fleming V, Somerset S. Group concept mapping to facilitate participatory design of the web-based pain-at-work toolkit2024. 5839–43 p.

[40] Abelson J, Tripp L, MacNeil M, Lang A, Fancott C, Ganann R, et al. Development of the Engage with Impact Toolkit: A comprehensive resource to support the evaluation of patient, family and caregiver engagement in health systems. Health Expectations. 2023;26(3):1255–65. doi: 10.1111/hex.13742 36942646 PMC10154848

[41] Goff AJ, De Oliveira Silva D, Ezzat AM, Crossley KM, Pazzinatto MF, Barton CJ. Co-design of the web-based ‘My Knee’ education and self-management toolkit for people with knee osteoarthritis. DIGITAL HEALTH. 2023;9:20552076231163810. doi: 10.1177/20552076231163810 37009308 PMC10052584

[42] Goff AJ, Donaldson A, de Oliveira Silva D, Crossley KM, Barton CJ. People With Knee Osteoarthritis Attending Physical Therapy Have Broad Education Needs and Prioritize Information About Surgery and Exercise: A Concept Mapping Study. Journal of Orthopaedic & Sports Physical Therapy. 2022;52(9):595–606. doi: 10.2519/jospt.2022.11089 . 35712751

[43] Ahmed SU, Humphreys S, Rivers C, Jeffrey M, Fourney DR. Traumatic spinal cord injuries among Aboriginal and non-Aboriginal populations of Saskatchewan: a prospective outcomes study. Can J Surg. 2020;63(3):E315–e20. Epub 2020/06/05. doi: 10.1503/cjs.012819 ; PubMed Central PMCID: PMC7829008. 32496034 PMC7829008

[44] Krause JS, Dismuke CE, Acuna J, Sligh-Conway C, Walker E, Washington K, et al. Race–ethnicity and poverty after spinal cord injury. Spinal Cord. 2014;52(2):133–8. doi: 10.1038/sc.2013.147 24296805 PMC3946286

[45] Furlan JC. Racial and Ethnical Discrepancies and Similarities in the Epidemiology, Survival, and Neurological Outcomes After Acute Traumatic Spinal Cord Injury: A Retrospective Cohort Study Using Data from the NASCIS-1 Trial. Top Spinal Cord Inj Rehabil. 2023;29(Suppl):88–102. Epub 2024/01/04. doi: 10.46292/sci23-00055S ; PubMed Central PMCID: PMC10759859. 38174140 PMC10759859

